# Role of 2-[^18^F]FDG-PET as a biomarker of upper motor neuron involvement in amyotrophic lateral sclerosis

**DOI:** 10.1007/s00415-025-13501-6

**Published:** 2025-11-17

**Authors:** Sara Cabras, Umberto Manera, Francesca Di Pede, Grazia Zocco, Rosario Vasta, Andrea Novara, Emilio Minerva, Enrico Matteoni, Filippo De Mattei, Giorgio Pellegrino, Maurizio Grassano, Barbara Iazzolino, Francesca Palumbo, Stefano Callegaro, Giulia Polverari, Silvia Daniela Morbelli, Matteo Pardini, Agostino Chiaravalloti, Orazio Schillaci, Klaus Leonard Leenders, Rosalie Vered Kogan, Cristina Moglia, Andrea Calvo, Adriano Chiò, Marco Pagani, Antonio Canosa

**Affiliations:** 1 ALS Centre, ‘Rita Levi Montalcini’ Department of Neuroscience, University of Turin, Via Cherasco 15, 10126 Turin, Italy; 2 School of Advanced Studies, Centre for Neuroscience, University of Camerino, Camerino, Italy; 3 Azienda Ospedaliero-Universitaria Città Della Salute e Della Scienza Di Torino, Neurology Unit 1U, Turin, Italy; 4 Positron Emission Tomography Centre AFFIDEA-IRMET S.P.A, Turin, Italy; 5 Department of Medical Sciences, University of Turin, Turin, Italy; 6 Azienda Ospedaliero-Universitaria Città Della Salute e Della Scienza Di Torino, Nuclear Medicine Unit, Turin, Italy; 7 Department of Neuroscience, Rehabilitation, Ophthalmology, Genetics, Maternal and Child Health (DINOGMI), University of Genoa, Genoa, Italy; 8 IRCCS Ospedale Policlinico San Martino, Genoa, Italy; 9 Department of Biomedicine and Prevention, University of Rome ‘Tor Vergata’, Rome, Italy; 10 IRCCS Neuromed, Pozzilli, Italy; 11 Department of Neurology, University of Groningen, University Medical Centre Groningen, Groningen, The Netherlands; 12 Department of Nuclear Medicine and Molecular Imaging, University of Groningen, University Medical Centre Groningen, Groningen, The Netherlands; 13 Neuroscience Institute of Turin (NIT), Turin, Italy; 14 Institute of Cognitive Sciences and Technologies, C.N.R., Rome, Italy; 15 Department of Medical Radiation Physics and Nuclear Medicine, Karolinska University Hospital, Stockholm, Sweden

**Keywords:** Amyotrophic lateral sclerosis, 2-[^18^F]FDG-PET, Upper motor neuron, Motor neuron disease

## Abstract

**Introduction:**

Amyotrophic Lateral Sclerosis (ALS) affects upper (UMN) and lower (LMN) motor neurons. ALS diagnosis is challenging, especially in predominant LMN phenotypes. Electromyography can disclose LMN damage, while UMN involvement is detectable by clinical examination, with possible support of magnetic resonance imaging (MRI) and transcranial magnetic stimulation. Our aim was to investigate the role of 2-[^18^F]FDG-PET as an UMN biomarker in ALS.

**Methods:**

In our cross-sectional study, we created an UMN burden score. Performing a multiple regression analysis in SPM12, we evaluated the relationship between UMNBS and brain metabolism. We split ALS cohort based on the UMN burden score median value (group A—under median, group B—above median). We ran a full factorial analysis including group A and B and healthy controls, followed by group comparisons.

**Results:**

We included 118 ALS patients (group A and B, *N* = 59), with a median UMN burden score of 9.50 and a left lateralization of UMN signs. We found a negative correlation between motor cortex metabolism and UMN burden score. Comparing each ALS group with healthy controls, we found relative hypometabolism in the left frontal lobe and relative bilateral, right-prevalent hypermetabolism of cerebellum and corticospinal tracts. The relative hypermetabolism in corticospinal tracts was more evident in the group with low UMN signs.

**Conclusions:**

Motor cortex metabolism reflects UMN burden. Corticospinal tracts’ metabolic changes could provide information about UMN involvement even in patients with predominant LMN phenotype, suggesting a possible role of brain 2-[^18^F]FDG-PET as an UMN biomarker in ALS patients.

**Supplementary Information:**

The online version contains supplementary material available at 10.1007/s00415-025-13501-6.

## Introduction

Amyotrophic Lateral Sclerosis (ALS) is a neurodegenerative disorder characterized by progressive motor decline associated with frontotemporal cognitive impairment in half of the patients [1]. By definition, ALS affects both upper (UMN) and lower (LMN) motor neurons, and is in the middle of the spectrum ranging from pure upper to pure lower motor neuron diseases [2]. Several peripheral neuromuscular disorders can mimic ALS, leading to misdiagnosis of ALS cases with predominant LMN signs, which can mask UMN clinical involvement. Therefore, there is a need to find UMN biomarkers to be translated in the clinical setting. While electromyography can detect the lower motor neuron dysfunction, the search for reliable tools to assess the upper motor neuron degeneration in the clinical routine is still underway. Transcranial magnetic stimulation can support diagnosis, providing evidence of upper motor neuron involvement [3], although available data are still inconclusive on its use in a clinical setting. Neurofilament light chain (NfL) has been proposed as a supportive UMN biomarker as well [3]. Despite the fact that recent papers [4, 5] displayed a correlation between NfL and UMN signs in ALS patients, pure UMN syndromes, such as Primary Lateral Sclerosis and Hereditary Spastic Paraplegia, showed low levels of them [6, 7]. Therefore, NfL likely represents a reliable marker of neurodegeneration rather than an UMN marker. As new disease-modifying therapies are emerging, there is a growing need for an upper motor neuron biomarker to accelerate the diagnostic process and, consequently, the inclusion of patients in clinical trials, and to follow over time the brain neurodegenerative process. Several papers investigated the role of advanced brain magnetic resonance imaging (MRI) techniques as UMN biomarkers. Multiparametric MRI seems to be the most promising tool in this field [8]. Quantitative susceptibility mapping (QSM) of the precentral cortex improved reliability of the motor band sign providing a quantitative measure for iron accumulation, even if QSM changes are detectable predominantly in UMN phenotypes, reducing the sensitivity of this technique [9]. Motor cortex thickness (MCT), diffusion tensor imaging (DTI), and fiber density (FD) measures of the corticospinal tracts showed good performances as UMN markers [10]. Nonetheless, values, especially in patients with predominant lower motor neuron signs, are not consistently different compared to healthy controls [10], suggesting that structural alterations are not always detectable, even if neuropathological findings of macrophagic activation along corticospinal tracts have been described in progressive muscular atrophy (PMA), a pure lower motor neuron disease [11]. Very few papers investigated the role of 2-[^18^F]FDG-PET in assessing the brain metabolic changes associated with upper motor neuron damage in the Motor Neuron Disease (MND) spectrum [12]. Notably, the previous studies on 2-[^18^F]FDG-PET demonstrated its usefulness in localizing brain areas involved in the neurodegenerative process [13, 14] and evaluating the extent of the pathological burden [15]. De Vocht et al. showed that 2-[^18^F]FDG-PET alterations in *C9orf72* presymptomatic carriers precede NfL raise in cerebrospinal fluid and clinical signs or cognitive test alterations, suggesting brain metabolism as an early biomarker of neurodegeneration [16]. 2-[^18^F]FDG-PET in other disease affecting UMN, as PLS [17], HSP [18], and corticobasal syndromes [19], displayed perirolandic hypometabolism with metabolic alterations reflecting the side and severity of motor clinical involvement.

Our study aimed at evaluating the role of brain 2-[^18^F]FDG-PET to assess upper motor neuron involvement in ALS patients. First, we investigated the association between UMN clinical signs and metabolic changes of brain 2-[^18^F]FDG-PET. Second, we looked for eventual brain 2-[^18^F]FDG-PET alterations in patients without UMN clinical signs, detecting a subclinical pyramidal involvement.

## Materials and methods

We conducted a cross-sectional retrospective observational study adhering to the relevant STROBE checklist.

### Participants

We included all consecutive patients diagnosed with ALS according to Gold Coast diagnostic criteria [3] between 2017 and 2021 at the ALS Centre of Turin, Italy, who underwent brain 2-[^18^F]FDG-PET at the time of diagnosis. The following demographic and clinical characteristics were collected: age at PET, sex, site of onset (spinal/ bulbar, upper/lower limbs, right-/left-sided involvement), presence of *SOD1*, *TARDBP*, *FUS* pathogenic variants and *C9orf72* GGGGCC hexanucleotide expansion, neuropsychological evaluation [20, 21], ALS Functional Rating Scale-Revised (ALSFRS-R) [22] at PET, calculated ΔALSFRS-R (defined as [(48-ALSFRS-R score at scan)/disease duration at scan (in months)])[15] and King’s stage at PET (calculated from the ALSFRS-R score [23] and combining King’s stages 4a and 4b as stage 4). The neuropsychological evaluation was performed as previously reported [24]. Briefly, patients were tested using the Edinburgh Cognitive and Behavioral ALS Screen (ECAS) [25], which explores multiple cognitive domains, including executive and visuospatial functions, language, fluency and memory, as well as behavioral changes in ALS patients, and additional tests according to the Diagnostic Criteria for the Behavioral variant of Frontotemporal Dementia [21], and ALS-FTD Consensus Criteria (ALSFTD-CC) [20]. Genetic analysis was performed as reported elsewhere [14].

We retrospectively collected data on UMN burden and atrophy distribution from medical records of our Centre, thus creating an UMN burden score (UMNBS) ranging from 0 to 24 (see Table 1). Brisk reflexes and retained reflexes in wasted limbs were scored as 1; otherwise, a 0 point score was assigned. We included jaw jerk (0 = absent, 1 = present, 2 = brisk), palmomental reflex (0 = absent, 1 = present), Hoffmann sign (0 = absent, 1 = present), and Babinski sign (0 = absent, 1 = present) as pathological reflexes. We rated limb spasticity using the Modified Ashworth Scale [26], converted as performed in the Penn Upper Motor Neuron Score (PUMNS) [27]. We collected data on clonus and emotional lability (absence/presence) when present, but they were excluded when calculating the UMNBS because of the large amount of missing data.

**Table 1:** UMNBS and atrophy score. PUMNS: Penn Upper Motor Neuron Score. MAS: Modified Ashworth Scale

UMNBS	
Limbs reflexes *Bicipital, brachioradialis, patellar, ankle*	0 if absent, low or normal in a normotrophic area
	1 if brisk or retained in an atrophic area
Pathological reflexes *Palmomental reflex (bilateral), Hoffmann’s sign (bilateral), Babinski’s sign (bilateral)*	0 if absent
	1 if present
Jaw jerk	0 if absent
	1 if present
	2 if brisk
Spasticity at four limbs (as PUMNS)	0 if normal muscle tone
	1 if MAS 2–3
	2 if MAS 4–5

Atrophy	
Proximal and distal upper and lower limbs atrophy	0 if normal
	1 if atrophy

For a more comprehensive evaluation of the metabolic changes associated with UMN involvement in ALS patients, we included 168 healthy controls (HC) collected from four centers (ALS Centre of Turin, Italy [14]; Nuclear Medicine Unit, Department of Health Sciences, University of Genoa, Italy [28]; Department of Biomedicine and Prevention, University of Rome Tor Vergata, Italy [29]; Department of Nuclear Medicine and Molecular Imaging, University Medical Center Groningen, University of Groningen, The Netherlands [28]) and presenting the following characteristics: (i) no history of neurological disorders and a normal neurological examination; (ii) brain PET scan reported as normal by the nuclear medicine physician.

### 2-[^18^F]FDG‑PET image acquisition and pre–processing

Brain 2-[^18^F]FDG-PET was performed according to published guidelines [30]. Patients fasted at least 6 h before the exam. Blood glucose was < 7.2 mmol/l in all cases before the procedure. After a 20-min rest, about 185 MBq of 2-[^18^F]FDG was injected. The acquisition started 60 min after the injection. Scanner details for each center are reported in Table 1 of Supplementary material [14, 28, 29, 31]. Brain CT and PET scan were sequentially acquired, the former being used for attenuation correction of PET data. The PET images were reconstructed with four iterations and 28 subsets with an initial voxel size of 2.34 × 2.34 × 2.00 mm, and data were collected in 128 × 128 matrices. Images were spatially normalized to a customized brain 2-[^18^F]FDG-PET template [32] and subsequently smoothed with a 10-mm filter in MATLAB R2018b (MathWorks, Natick, MA, USA). Intensity normalization was performed at individual level averaging each voxel for the mean value of the whole brain.

### Statistical analysis

The demographic and clinical characteristics of patients and HC were analyzed using χ^2^ or Fisher’s exact test and t-student or Mann–Whitney test when appropriate. We used a log-rank test to compare survival between groups. Cronbach's alpha and McDonald’s omega were calculated as measures of internal consistency of the UMN burden score derived from our template. The analyses were performed using the software jamovi (The jamovi project, 2022, version 2.3) with *p*-value < 0.05 taken as significant. SPSS (IBM SPSS statistics version 28) was employed for graphical representations.

As regards the analyses of PET data, to evaluate the relationship between the UMNBS and brain metabolism in the ALS cohort, we performed a multiple regression analysis using SPM12 implemented in MATLAB R2022a, including the following covariates: age at the time of PET, sex, site of onset (spinal/bulbar), cognitive status, and ALS Functional Rating Scale-Revised (ALSFRS-R) at PET. Cognitive status was classified as cognitively normal (ALS-CN), ALS with cognitive impairment (ALSci), ALS with behavioral impairment (ALSbi), ALS with cognitive and behavioral impairment (ALScbi), ALS with Frontotemporal Dementia (ALS-FTD), according to published diagnostic criteria [20].

Then, we compared ALS patients with HC using the two-sample t test model of SPM12. Subsequently, we split the ALS cohort in two groups based on the median value of UMNBS (group A—under median value and group B—above median value) to further characterize the brain metabolic correlates of the UMNBS. The full factorial design as implemented in SPM12 was employed to test the hypothesis that differences among groups (group A, B and HC) exist overall (i.e., main effect of groups). In case the hypothesis was confirmed, we used the two-sample t test model of SPM12 to compare groups A and B between each other and with HC. Age at PET, sex, and center of origin were used as covariates in all the analyses including HC; the comparison between the two ALS groups was adjusted also for site of onset, cognitive category, and ALSFRS-R. In all the analyses, the height threshold was set at *p* < 0.001 (*p* < 0.05 FWE-corrected at cluster level). Only clusters containing > 125 contiguous voxels were considered significant. Brodmann areas (BAs) were identified at a 0–2-mm range from the Talairach coordinates of the SPM output isocentres corrected by Talairach Client (http://www.talairach.org/index.html).

## Results

### Demographic and clinical data

Out of 277 potentially eligible subjects, 118 were included. Patients excluded for missing data on UMNBS and covariates did not differ from included cases for clinical and demographic characteristics, except for a significant difference in disease duration at PET and ∆ALSFRS-R (data not shown). Demographic and clinical characteristics of the ALS cohort are reported in Table 2. Group A and B included 59 patients each and showed no statistically significant differences in sex, age at onset, age at PET, total ALSFRS-R score, ∆ALSFRS-R, cognitive status, and *C9orf72* expansion. As compared to Group B, Group A included a higher number of spinal onset patients and showed a longer diagnostic delay, without reaching the statistical significance. No differences in survival were found between the two groups (*p* = 0.93, Fig. 1 of Supplementary Material). UMNBS was normally distributed in our cohort, ranging from 1 to 22 (Fig. 2 of Supplementary Material). Almost all patients presented with hyperreflexia (*n* = 117, 99%), while a smaller percentage had Babinski sign (*n* = 35, 30%) and spasticity (*n* = 26, 22%). No differences between ALS and HC in demographic features were found (Table 2 of Supplementary Material).

**Table 2:** Demographic and clinical characteristics of ALS cohort. ALS-CN: cognitively normal ALS. ALSbi: ALS with behavioral impairment. ALSci: ALS with cognitive impairment. ALScbi: ALS with cognitive and behavioral impairment. ALS-FTD: ALS with frontotemporal dementia

	Total	Group A	Group B	*p* value
N (%)	118 (100)	59 (50)	59 (50)	
Sex (M, %)	72 (61)	37 (62.7)	35 (59.3)	0.706
Age at onset, years; mean (SD)	61.0 (12.0)	62.48 (10.2)	59.52 (13.5)	0.186
Age at PET, years; mean (SD)	61.9 (12.1)	63.53 (10.2)	60.30 (13.6)	0.157
Disease duration at PET, months; mean (SD)	13.2 (8.57)	14.65 (9.7)	11.78 (7)	0.069
Diagnosis delay, months; mean (SD)	10.5 (7.91)	11.78 (8.68)	9.305 (6.91)	0.08
∆ALSFRS-r; mean (SD)	0.986 (0.821)	0.961 (0.9)	1.01 (0.7)	0.743
Total AlSFRS-r score at PET; mean (SD)	38.7 (5.28)	38.48 (5.8)	38.93 (4.8)	0.640
Site of onset				0.134
Bulbar (N, %)	29 (24.6)	11 (18.6)	18 (30.5)	
Spinal (N, %)	89 (75.4)	48 (81.4)	41 (69.5)	
Cognitive (N, %)	0 (0)	0 (0)	0 (0)	
Cognitive impairment				0.675
CN (N, %)	75 (63.6)	39 (66.1)	36 (61.0)	
ALSbi (N, %)	7 (5.9)	3 (5.1)	4 (6.8)	
ALSci (N, %)	28 (23.7)	15 (25.4)	13 (22.0)	
ALScbi (N, %)	7 (5.9)	2 (3.4)	5 (8.5)	
FTD (N, %)	1 (0.8)	0 (0)	1 (1.7)	
Presence of *C9orf72* expansion (N, %)	7 (6.4)	3 (5.6)	4 (7.1)	1.00

**Fig. 1:**
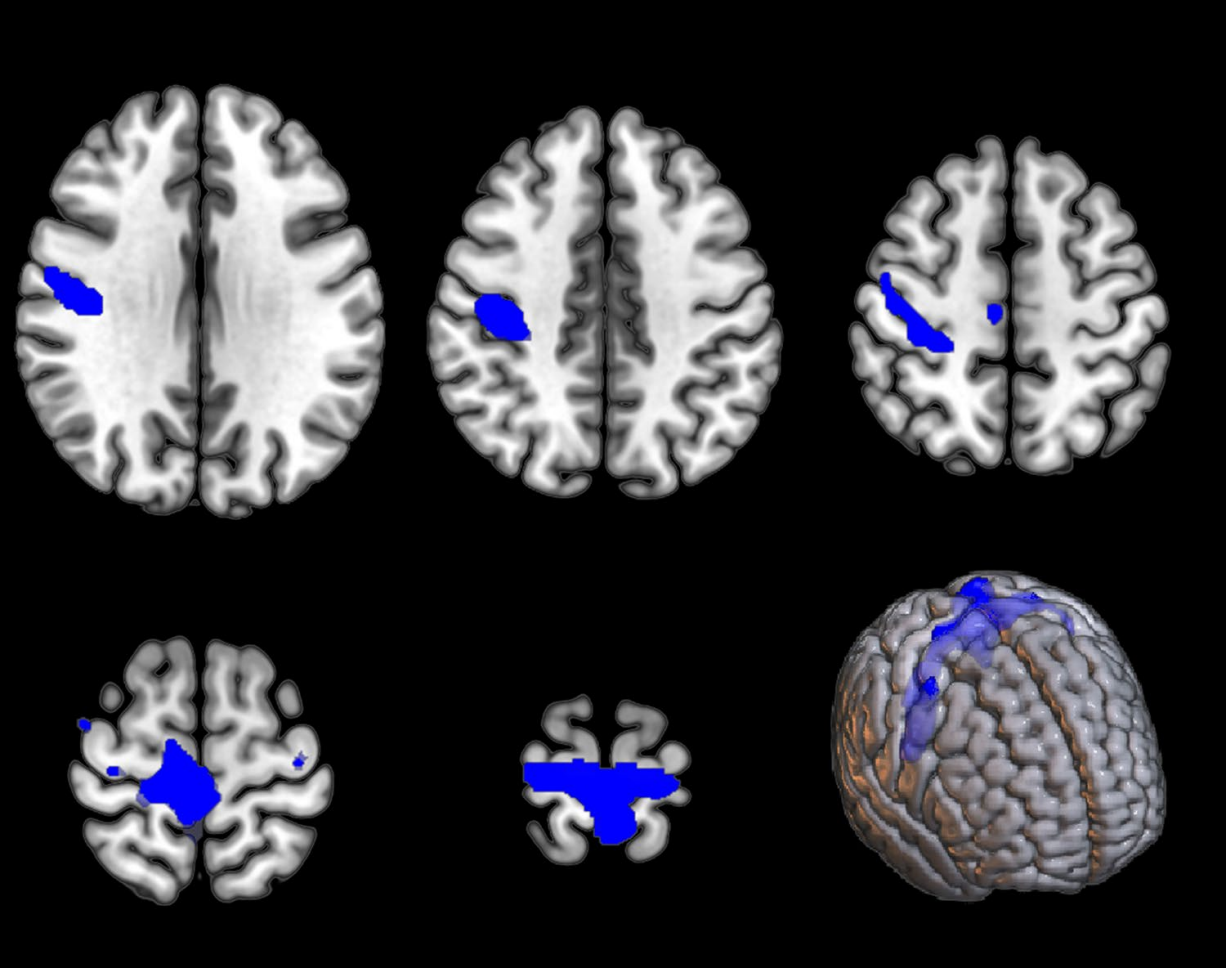
Clusters resulting from the negative correlation between whole-brain metabolism and UMNBS in the ALS cohort are marked in blue and are reported on axial sections of a brain magnetic resonance imaging template and on the brain surface of a glass brain rendering (bottom right)

**Fig. 2:**
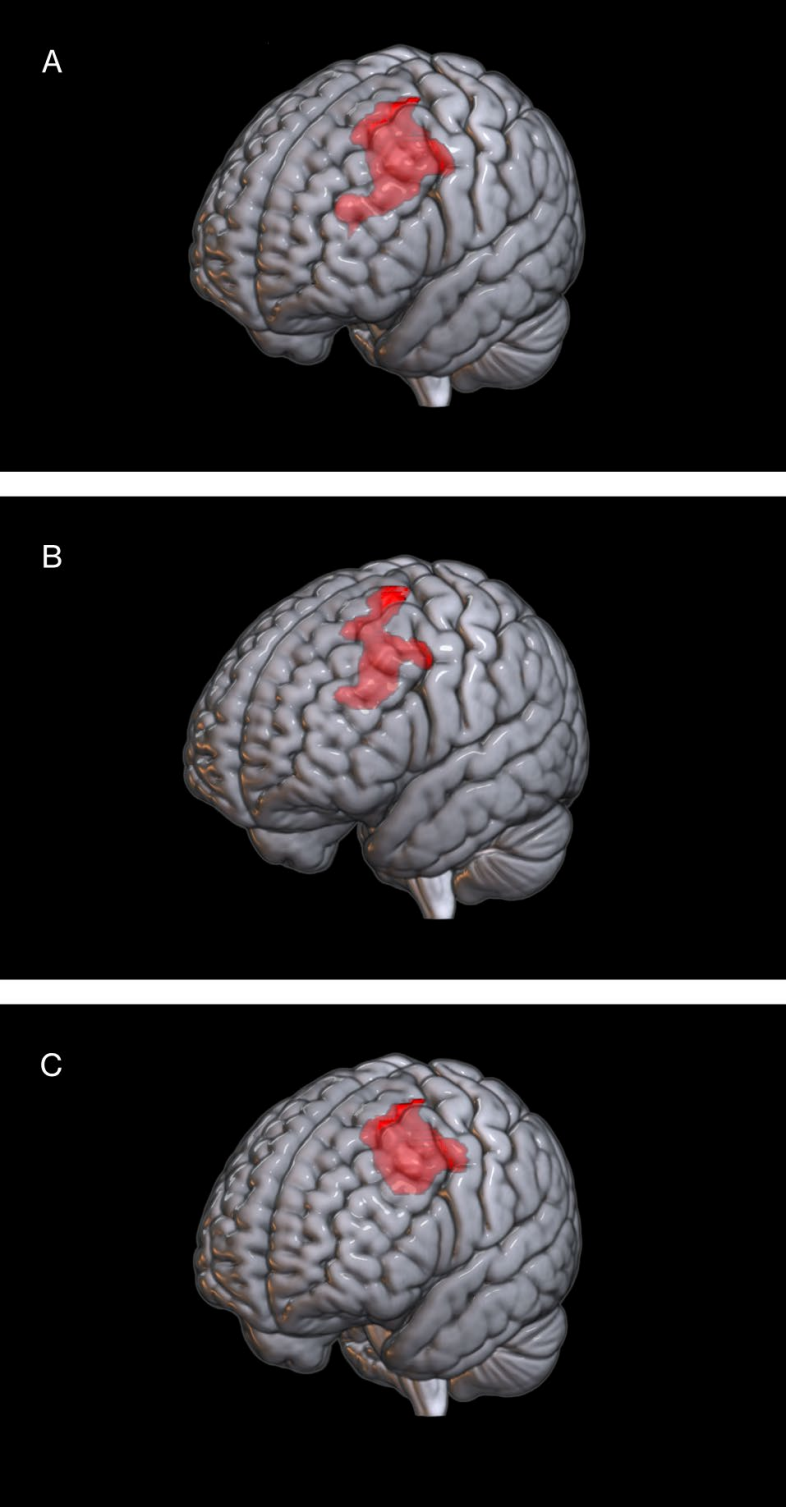
Comparison with HC. Clusters of relative hypometabolism of ALS patients (section A, *N* = 118), Group A (section B, *N* = 59), and Group B (section C, *N* = 59), respectively. Clusters are marked in red and are reported on the brain surface of a glass brain rendering

### Validity of UMNBS

Nineteen elements were included in the UMNBS (Table 1), with a Cronbach’s alpha value of 0.859 and a McDonald’s omega of 0.867.

### Correlation between UMNBS and brain metabolism in the whole ALS cohort

Multiple regression analysis showed a statistically significant negative correlation between the UMNBS and brain metabolism in bilateral precentral and postcentral gyrus and right medial frontal gyrus (Fig. 1, Table 3).

**Table 3:** Clusters resulting from the negative correlation between whole-brain metabolism and UMNBS in the ALS cohort (BA, Brodmann area)

*p* (FWE-corrected)	*p* (FDR-corrected)	Cluster extent	Z-score	Talairach coordinates (x,y,z)			Lobe	Cortical region	BA
0.000	0.000	2846	5.56	48.0	− 10.0	28.0	Frontal	Right precentral gyrus	6
			5.48	6.0	− 26.0	68.0	Frontal	Right medial frontal gyrus	6
			4.99	40.0	− 18.0	36.0	Frontal	Right precentral gyrus	4
			4.10	− 20.0	− 24.0	71.0	Frontal	Left precentral gyrus	4
			3.94	24.0	− 30.0	55.0	Parietal	Right postcentral gyrus	3
			3.84	− 6.0	− 39.0	72.0	Parietal	Left postcentral gyrus	5
			3.65	44.0	− 3.0	55.0	Frontal	Right precentral gyrus	6

### Comparison of brain metabolism among ALS groups and HC

**Fig. 3:**
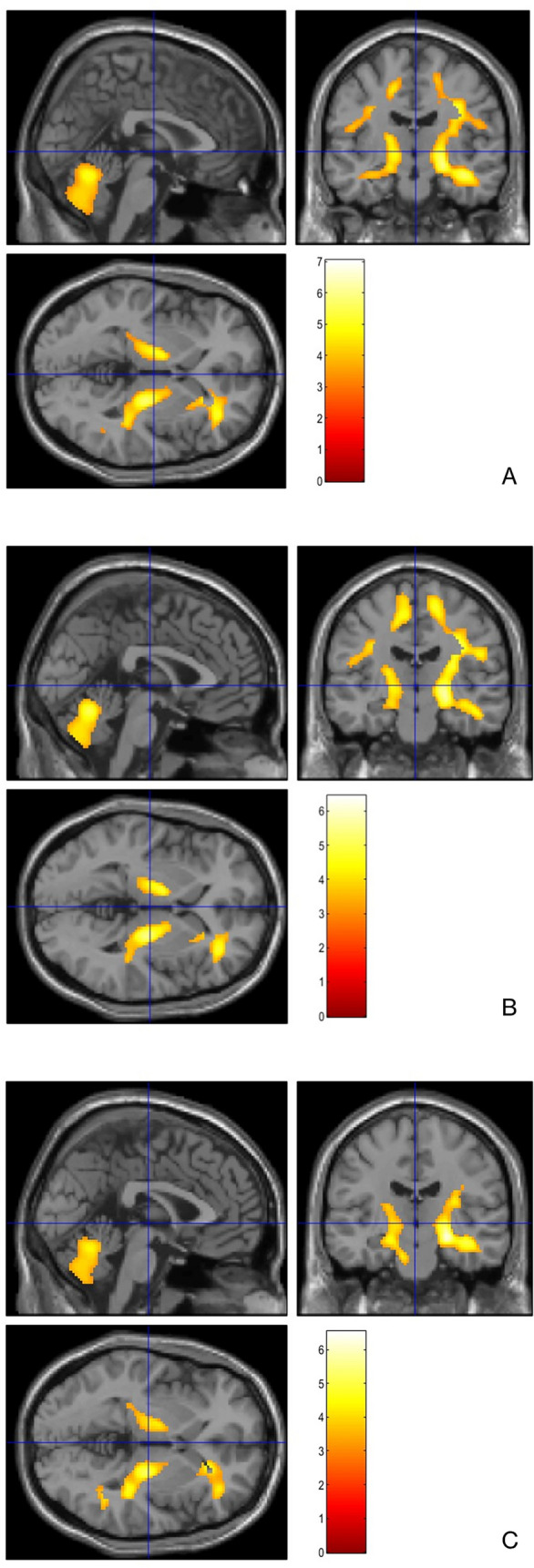
Comparison with HC. Clusters of relative hypermetabolism of ALS patients (section A, *N* = 118), group A (section B, *N* = 59), and group B (section C, *N* = 59). They are represented on a brain magnetic resonance imaging (MRI) template

Comparison between the whole cohort of ALS and HC displayed a significant relative hypometabolism in the left frontal lobe (precentral, superior and middle frontal gyrus; Fig. 2A, Table 4) and relative hypermetabolism in bilateral cerebellar hemispheres and corticospinal tracts (CST) (Fig. 3A) in ALS cases.

**Table 4:** Clusters of relative hypometabolism resulting in the whole ALS cohort compared to HC (BA, Brodmann area)

*p* (FWE-corrected)	*p* (FDR-corrected)	Cluster extent	Z-score	Talairach coordinates (x,y,z)			Lobe	Cortical region	BA
0.000	0.002	1402	5.10	− 40.0	23.0	39.0	Frontal	Left Precentral Gyrus	9
			4.36	− 50.0	8.0	42.0	Frontal	Left Middle Frontal Gyrus	6
			4.29	− 26.0	22.0	50.0	Frontal	Left Superior Frontal Gyrus	8
			3.75	− 36.0	40.0	24.0	Frontal	Left Middle Frontal Gyrus	10
			3.63	− 40.0	30.0	24.0	Frontal	Left Middle Frontal Gyrus	46

The full factorial design resulted in a significant main effect of groups (data not shown); therefore, we performed the post hoc comparisons among groups.

We explored the difference between group A and HC, resulting in a relative hypometabolism in left precentral, middle, and superior frontal gyri (Fig. 2B, Table 5) and a hypermetabolism in CST and cerebellum bilaterally (Fig. 3B).

**Table 5:** Clusters of relative hypometabolism in group A compared to HC (BA, Brodmann area)

*p* (FWE-corrected)	*p* (FDR-corrected)	Cluster extent	Z-score	Talairach coordinates			Lobe	Cortical region	BA
0.004	0.015	904	4.61	− 38.0	25.0	37.0	Frontal	Left Precentral Gyrus	9
			3.94	− 26.0	7.0	62.0	Frontal	Left Middle Frontal Gyrus	6
			3.88	− 40.0	28.0	24.0	Frontal	Left Middle Frontal Gyrus	46
			3.83	− 48.0	10.0	42.0	Frontal	Left Middle Frontal Gyrus	8
			3.79	− 24.0	24.0	50.0	Frontal	Left Superior Frontal Gyrus	8

When a comparison between group B and HC was performed, a relative hypometabolism in left middle and superior frontal gyri (Fig. 2C, Table 6) and a less pronounced hypermetabolism in CST and cerebellum bilaterally were found (Fig. 3C).

**Table 6:** Clusters of relative hypometabolism in group B compared to HC (BA, Brodmann area)

*p* (FWE-corrected)	*p* (FDR-corrected)	Cluster extent	Z-score	Talairach coordinates (x,y,z)			Lobe	Cortical region	BA
0.001	0.004	1279	4.96	− 38.0	23.0	41.0	Frontal	Left Middle Frontal Gyrus	8
			4.36	− 28.0	22.0	50.0	Frontal	Left Superior Frontal Gyrus	8
			4.17	− 50.0	4.0	42.0	Frontal	Left Middle Frontal Gyrus	6

Moreover, we detected a significant relative hypometabolism in group B compared to group A in bilateral precentral gyrus and right medial frontal gyrus (Supplementary Material, Fig. 3 and Table 3).

We checked for lateralization of UMN burden in our cohort, finding significantly higher UMNBS on the left side compared to the right side (*p* = 0.039).

## Discussion

Our study aimed at evaluating brain 2-[^18^F]FDG-PET as an UMN biomarker in ALS. As stated by Henderson and Devine, an UMN biomarker requires correlation with progression rate, low subjectivity, and inter-rater variability, and should account for the heterogeneity of ALS phenotypes [8]. One of the strengths of our study is that it overcomes subjectivity and inter-rater variability, since the semi-quantitative analyses of 2-[^18^F]FDG-PET data are rater-independent. Brain metabolism has been already demonstrated to be related to disease staging and survival of ALS patients [15, 33] and 2-[^18^F]FDG-PET has been proven to localize different cortical involvement in distinct phenotypes of the disease [13, 34].

The need of UMN markers to reduce the time to diagnosis has already been suggested by neurophysiological studies investigating the role of cortical hyperexcitability in predominant lower motor neuron ALS phenotypes [35]. Indeed, in our cohort, the group with a lower UMN burden (group A) displayed a trend toward a longer diagnostic delay compared to group B. A recent paper investigating brain metabolic changes as a possible UMN biomarker and their correlation with motor-evoked potential, found that the two techniques identified UMN involvement at a group level in accordance with each other [36].

We retrospectively created an UMN burden score based on available data of our patients. We did not use other UMN scales available in literature (Penn UMN score [37] and MGH score [38]), since we were not able to obtain these total scores from retrospective data due to the high number of missing values. Nevertheless, the UMNBS structure is similar to the MGH score, which displayed a good correlation to neuroimaging findings as the Penn UMN score [39]. In our score, we excluded pseudobulbar affect for the following reasons: first, it could be masked by therapy with serotoninergic antidepressants; second, there was a large amount of missing data; third, it seems scarcely related to UMN burden and imaging biomarkers [39]. In keeping with published studies [39, 40], we confirmed a relatively low frequency of spasticity in our cohort, but we included it in the scale as it is a definite hallmark of UMN involvement and it added value to UMNBS performance.

We observed a decrease in cortical metabolism, including Brodmann Area (BA) 4, as UMNBS values increased. This is consistent with the previous MRI studies showing that motor cortex damage parallels UMN clinical involvement [10, 39, 41]. Our results point out that the UMNBS employed in this study properly reflects the cortical UMN damage, confirming both the reliability of the scale in describing UMN involvement and the role of 2-[^18^F]FDG-PET to disclose the neurodegenerative process underlying ALS. Moreover, we explored regions that could be altered in ALS patients with low UMN clinical burden, evaluating areas deriving from the comparison between ALS patients and HC. We found no differences between ALS patients (i.e., the whole group and groups A and B separately) and HC in the primary motor cortex. A possible explanation could be the co-existence of increased metabolism due to astrogliosis and microglial activation [39, 41, 42] and a relative hypometabolism, deriving from neuronal loss. The prevalence of one of these opposite phenomena might account for a significant difference in the direct comparison between group A and B. Accordingly, in a previous study, we found that *SOD1*-ALS patients, who typically have low clinical UMN burden, showed motor cortex relative hypermetabolism compared to HC, while relative hypometabolism in the same regions was found in sporadic ALS compared to *SOD1* carriers [14, 43].

The metabolic differences extending to prefrontal regions in ALS patients compared to HC have been widely reported and could be likely attributed to cognitive changes [15].

In the comparison of ALS cases with HC, the relative hypermetabolism in CST and the cerebellum is in agreement with the previous studies [44, 45]. CST relative hypermetabolism was more pronounced on the right side, reflecting the left-sided prevalence of UMN signs. A possible explanation of this finding resides on neuroinflammation and astrogliosis surrounding motor neurons axons, as suggested by literature showing subcortical hypermetabolism in ALS [46, 47] and macrophage accumulation along CST [11]. Cerebellar hypermetabolism has been previously interpreted as a compensatory hyperactivity of preserved circuits [33] or an early cerebellar involvement, as suggested by neuropathological data from Brettschneider and colleagues [48]. We found no statistically significant correlation between UMNBS and CST metabolism in the regression analysis. Interestingly, in the comparison with HC, group A showed a larger cluster of relative hypermetabolism in CST and cerebellum compared to group B, although not reaching statistical significance in the direct comparison between the two ALS groups. MRI studies showed a correlation between CST structural changes and UMN clinical burden. Nitert et al. found differences in CST fiber density between healthy controls and patients with high UMN clinical burden, but not patients with low UMN signs [10]. Other studies found a reduction of fractional anisotropy of CST with increasing UMN involvement [49] and with disease progression [50, 51]. On the contrary, we hypothesize that CST relative hypermetabolism is present independently of the clinical UMN signs, thus representing a marker of UMN involvement across the spectrum of ALS phenotypes.

Our study is not without limitations. First, the cross-sectional design of our study does not allow to assess longitudinal changes that could disentangle the debate about the ‘dying-back’ and ‘dying-forward’ UMN damage in ALS. Second, the sample size could have limited our power to disclose mild metabolic differences. Third, we used our UMN score, which has not been validated before in literature. Finally, we do not have MRI and neurophysiological data for an adequate number of subjects to allow further comparative analysis on CST integrity.

In conclusion, our data support the use of motor cortex and CST metabolism to evaluate UMN involvement in ALS, but further studies with longitudinal design and implementation of MRI metrics are necessary to pave the way from a group level toward a single-subject use of neuroimaging biomarkers in the evaluation of UMN damage.

## Supplementary Information

Below is the link to the electronic supplementary material.

Supplementary file1 (DOCX 293 KB)

## Data Availability

The data that support the findings of this study are available on request from the corresponding author. The data are not publicly available due to privacy or ethical restrictions.
