# Supplementary material for: Role of 2-[18F]FDG-PET as a biomarker of upper motor neuron involvement in amyotrophic lateral sclerosis
Source: J Neurol. 2025 Nov 17;272(12):766. doi: 10.1007/s00415-025-13501-6 (PMC12628406; doi:10.1007/s00415-025-13501-6)
Supplement: Supplementary file 1 — Supplementary file1 (DOCX 293 KB) [file 415_2025_13501_MOESM1_ESM.docx]

# **Role of 2-[^18^F]FDG-PET as a biomarker of upper motor neuron involvement in Amyotrophic Lateral Sclerosis**

Sara Cabras, MD^1,2^, Umberto Manera, MD, PhD^1,3^, Francesca Di Pede, MD^1^, Grazia Zocco, MD^1^, Rosario Vasta, MD, PhD^1^, Andrea Novara, MD^1^, Emilio Minerva, MD^1^, Enrico Matteoni, MD^1^, Filippo De Mattei, MD^1^, Giorgio Pellegrino, MD^1^, Maurizio Grassano, MD, PhD^1^, Barbara Iazzolino, MD, PhD^1^, Francesca Palumbo, MD^1^, Stefano Callegaro, MSc^1^, Giulia Polverari, MD, PhD^4^, Silvia Daniela Morbelli, MD, PhD^5,6^, Matteo Pardini, MD, PhD^7,8^, Agostino Chiaravalloti, MD, PhD^9,10^, Orazio Schillaci, MD^9^, Klaus Leonard Leenders, MD, PhD^11,12^, Rosalie Vered Kogan, MD^12^, Cristina Moglia, MD, PhD^1,3^, Andrea Calvo, MD, PhD^1,3,13^, Adriano Chiò, MD^1,3,13,14^*, Marco Pagani, MD, PhD^14,15^*, Antonio Canosa, MD, PhD^1,3,13,14^*

^1^ALS Centre, ‘Rita Levi Montalcini’ Department of Neuroscience, University of Turin, Turin, Italy

^2^School of Advanced Studies, Centre for Neuroscience, University of Camerino, Camerino, Italy

^3^Azienda Ospedaliero-Universitaria Città della Salute e della Scienza di Torino, Neurology Unit 1U, Turin, Italy

^4^Positron Emission Tomography Centre AFFIDEA-IRMET S.p.A., Turin, Italy

^5^Department of Medical Sciences, University of Turin, Turin, Italy

^6^Azienda Ospedaliero-Universitaria Città della Salute e della Scienza di Torino, Nuclear Medicine Unit, Turin, Italy

^7^Department of Neuroscience, Rehabilitation, Ophthalmology, Genetics, Maternal and Child Health (DINOGMI), University of Genoa, Genoa, Italy

^8^IRCCS Ospedale Policlinico San Martino, Genoa, Italy

^9^Department of Biomedicine and Prevention, University of Rome ‘Tor Vergata’, Rome, Italy

^10^IRCCS Neuromed, Pozzilli, Italy

^11^Department of Neurology, University of Groningen, University Medical Centre Groningen, Groningen, the Netherlands

^12^Department of Nuclear Medicine and Molecular Imaging, University of Groningen, University Medical Centre Groningen, Groningen, the Netherlands

^13^Neuroscience Institute of Turin (NIT), Turin, Italy

^14^Institute of Cognitive Sciences and Technologies, C.N.R., Rome, Italy

^15^Department of Medical Radiation Physics and Nuclear Medicine, Karolinska University Hospital, Stockholm, Sweden

*These authors equally contributed to the manuscript.

**Corresponding author**

Sara Cabras

ALS Centre, ‘Rita Levi Montalcini’ Department of Neuroscience, University of Turin; via Cherasco 15, 10126 Torino, Italy

sara.cabras@unito.it; phone number +39(0)116335439; fax number +39(0)116336454

ORCID 0000-0002-4988-2855.

**Supplementary Table 1.** Scanner details for the centres providing healthy controls.

| **Centre** | **Scanner** |
| --- | --- |
| ALS Centre of Turin, Italy | Discovery ST-E system (General Electric) |
| Nuclear Medicine Unit, Department of Health Sciences, University of Genoa, Italy | Siemens Biograph 16 PET/CT system (EANM Research Ltd-certified scanner) |
| Department of Biomedicine and Prevention, University of Rome Tor Vergata, Italy | Discovery VCT system (GE Medical Systems, Tennessee, USA) |
| Department of Nuclear Medicine and Molecular Imaging, University Medical Centre Groningen, University of Groningen, The Netherlands | Siemens Biograph mCT-64 PET/CT system |

|  |  |  |  |  |
| --- | --- | --- | --- | --- |
|  |  |  |  |  |
|  |  |  |  |  |
|  |  |  |  |  |
|  |  |  |  |  |
|  |  |  |  |  |
|  |  |  |  |  |
|  |  |  |  |  |

**Supplementary Figure 1.** Survival plots of group A (blue continuous line) compared to group B (red dashed line).

**
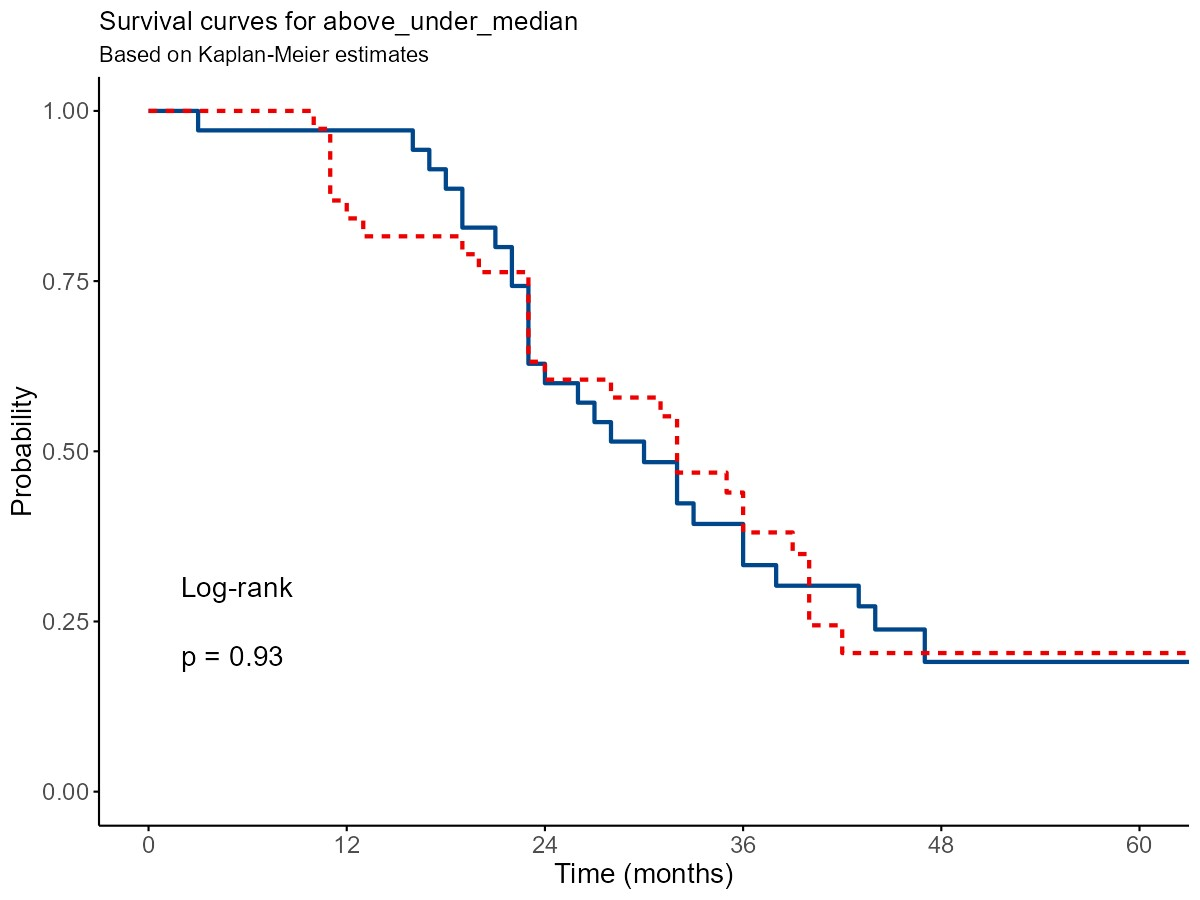
**

**Supplementary Figure 2.** Total frequency of UMNBS in ALS cohort (N=118).

**
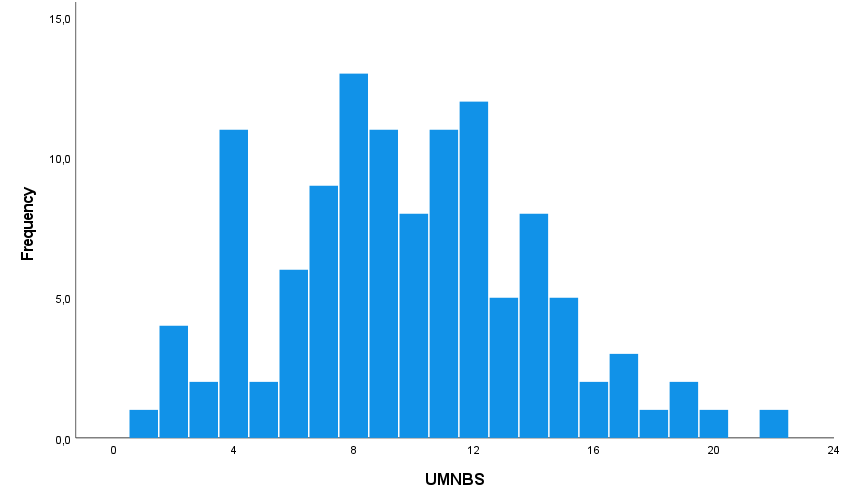
**

**Supplementary Table 2.** Comparison of demographic characteristics of ALS and HC

|  | Total | ALS | HC | p-value |
| --- | --- | --- | --- | --- |
| N (%) | 286 (100) | 118 (41) | 168 (59) |  |
| Sex (M, %) | 156 (55) | 72 (61) | 84 (50) | 0.065 |
| Age at PET, years; mean (SD) | 63.0 (13.8) | 63.5 (12) | 62.0 (14.8) | 0.196 |

**Supplementary Figure 3.** Clusters of relative hypometabolism in group B compared to group A are marked in yellow and are reported on axial sections of a brain magnetic resonance imaging template and on the brain surface of a glass brain rendering (bottom right).


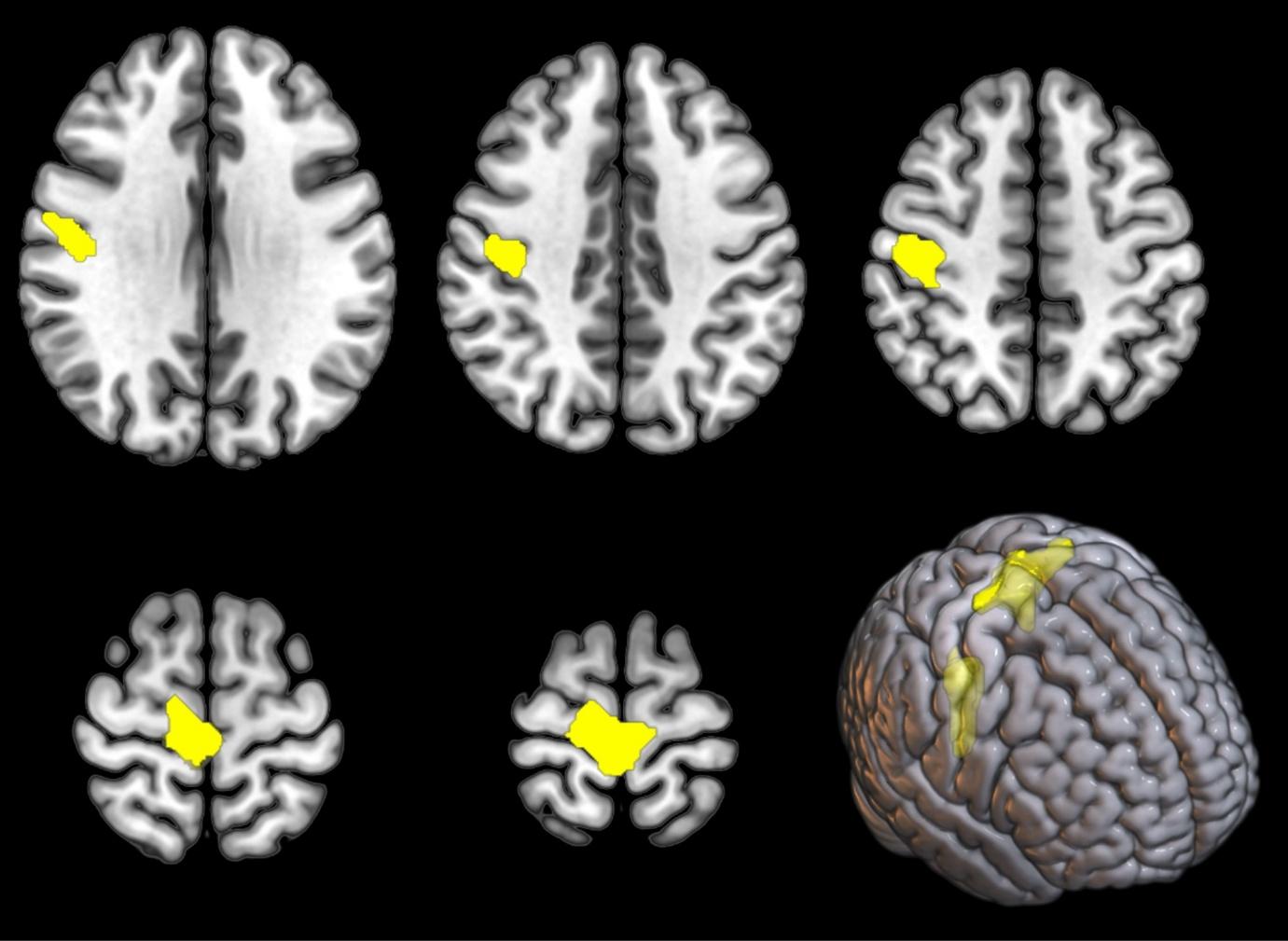


**Supplementary Table 3.** Clusters of relative hypometabolism in group B compared to group A (BA, Brodmann area).

| *p* (FWE-corrected) | *p* (FDR-corrected) | Cluster extent | Z-score | Talairach coordinates (x,y,z) | | | Lobe | Cortical region | BA |
| --- | --- | --- | --- | --- | --- | --- | --- | --- | --- |
| 0.002 | 0.005 | 973 | 4.90 | 6.0 | -24.0 | 68.0 | Frontal | Right Medial Frontal Gyrus | 6 |
|  |  |  | 3.94 | -18.0 | -24.0 | 71.0 | Frontal | Left Precentral Gyrus | 4 |
| 0.005 | 0.009 | 762 | 4.67 | 50.0 | -10.0 | 28.0 | Frontal | Right Precentral Gyrus | 6 |
|  |  |  | 4.60 | 42.0 | -15.0 | 41.0 | Frontal | Right Precentral Gyrus | 4 |
